# Three dimensional reconstruction of the mouse cerebellum in Hedgehog-driven medulloblastoma models to identify Norrin-dependent effects on preneoplasia

**DOI:** 10.1038/s42003-022-03507-5

**Published:** 2022-06-09

**Authors:** Nenad T. Pokrajac, Akshay Gurdita, Nobuhiko Tachibana, Nicholas J. A. Tokarew, Valerie A. Wallace

**Affiliations:** 1 Donald K. Johnson Eye Institute, Krembil Research Institute, University Health Network, Toronto, ON M5T 2S8 Canada; 2 Department of Laboratory Medicine and Pathobiology, University of Toronto, Toronto, ON M5S 1A8 Canada; 3 Department of Ophthalmology and Vision Sciences, University of Toronto, Toronto, ON M5T 3A9 Canada

**Keywords:** Cancer microenvironment, Disease model, 3-D reconstruction

## Abstract

Spontaneous mouse models of medulloblastoma (MB) offer a tractable system to study malignant progression in the brain. Mouse Sonic Hedgehog (Shh)-MB tumours first appear at postnatal stages as preneoplastic changes on the surface of the cerebellum, the external granule layer (EGL). Here we compared traditional histology and 3DISCO tissue clearing in combination with light sheet fluorescence microscopy (LSFM) to identify and quantify preneoplastic changes induced by disrupting stromal Norrin/Frizzled 4 (Fzd4) signalling, a potent tumour inhibitory signal in two mouse models of spontaneous Shh-MB. We show that 3DISCO-LSFM is as accurate as traditional histology for detecting Norrin/Fzd4-associated changes in PNL formation in *Ptch*^*+/−*^ mice and EGL hyperplasia in *Neurod2-SmoA1*^*+/−*^ mice. Moreover, we show that the anti-tumour effect of Norrin/Fzd4 signalling is restricted to the posterior region of the cerebellum and is characterized by defective neural progenitor migration away from the EGL. In conclusion, 3DISCO-LSFM is a valid way to monitor tumour initiation events in mouse MB models and reveals an unanticipated regional restriction of stromal signalling in constraining tumour initiation.

## Abstract (web summary)

Light sheet fluorescent microscopy can successfully be used to quantify preneoplastic alteration of tissue in mouse medulloblastoma, providing an alternative approach for 3D analysis of tumour initiation events in mice.

## Introduction

Tumour formation in epithelia follows a stepwise sequence where normal cells progress through a pre-neoplastic stage before becoming fully malignant^1^. Signals exchanged between pre-neoplastic cells and the surrounding microenvironment are vital in the tumour progression process, and influence whether pre-tumour cells are eliminated, rendered indolent or progress to malignancy^2^. Our understanding of this process in human brain tumourigenesis is incomplete because patient-derived tumour samples represent late stages of disease. Genetically engineered mouse models for the cerebellar tumour medulloblastoma (MB), the most common malignant brain tumour in children^3^, have provided key insights into early events in brain tumour progression^4^.

Sonic hedgehog (Shh)-MB, one of four molecularly distinct MB subtypes^5^, is characterized by hyperactivation of the Hedgehog morphogen signalling pathway. During postnatal cerebellar development, granule neuron progenitors (GNPs), the cell of origin for Shh-MB, undergo Shh-induced proliferation in the external granule layer (EGL) on the surface of the cerebellum^6–8^ followed by cell cycle exit, differentiation and migration to the internal granule layer to function as mature granule neurons^9,10^. Shh-MB is modelled in mice with haploinsufficiency for tumour suppressor genes (*Ptch*^*+/−*^)^11^ or transgenic expression of oncogenic activators (*NeuroD2-Smo*^*A1*^)^12^ of the Hh pathway. Preneoplastic lesion (PNL) formation and EGL hyperplasia are early hallmarks of the tumourigenic process in *Ptch*^*+/−*^ and *Neurod2-Smo*^*A1+/−*^ mouse models, respectively, underscoring the importance of characterizing the cellular and molecular events at this stage to gain deeper insight into tumourigenic progression^12,13^. All *Ptch*^*+/−*^ mice develop PNLs, defined as ectopic accumulations of GNPs that persist in the EGL beyond the period of normal GNP proliferation^13^, and tumour incidence directly correlates with the degree of PNL formation^14,15^. However, only a minority of PNLs progress to malignancy^16^. PNL formation and progression in *Ptch*^*+/−*^ mice are regulated by a variety of processes intrinsic to GNPs, including DNA damage, proliferation^17–20^ differentiation and migration^21^ and evasion of senescence^22^. In *Neurod2-Smo*^*A1+/−*^ mice the ubiquitous expression of the *NeuroD2-Smo*^*A1*^ transgene in GNPs induces EGL hyperplasia, instead of sporadic PNLs, however, manipulations that affect tumour progression in this model affect EGL hyperplasia^23^. Recently, we demonstrated that Norrin/Frizzled4 (Fzd4) signalling in the endothelium is a potent modulator of tumourigenesis in *Ptch*^*+/−*^ and *Neurod2-Smo*^*A1+/−*^ mice, identifying a critical role for the stroma during the earliest steps of tumourigenesis in the cerebellum^24^. Norrin, a secreted atypical Wnt ligand encoded by the *Ndp* gene, and its receptor Fzd4 play a well-described role in establishment and maintenance of blood brain barrier integrity in several regions of the central nervous system, including the cerebellum^23,25^. Genetic or acute antibody-mediated inhibition of Norrin/Fzd4 signalling in the endothelial cell compartment promotes MB progression in *Ptch*^*+/−*^ and *Neurod2-Smo*^*A1+/−*^ mouse models^24^, and is associated with enhanced PNL formation in the *Ptch*^*+/−*^ model.

The traditional histological approach to characterize preneoplasia involves confocal or brightfield imaging of tissue sections stained with nuclear and protein dyes, primarily Haemotoxylin and Eosin^13,26^. Tissue processing artifacts, including loss of sections and tissue distortion, can result in erroneous assignment of PNLs and volumetric estimations from 2D images can be inaccurate. In addition, PNLs form sporadically across the cerebellum, requiring sampling from large tissue volumes. Finally, tissue reconstruction from histological sections is laborious and low throughput, creating a significant barrier for timely and efficient specimen analysis, and for the detection and quantification of subtle or regionally restricted phenotypes.

The establishment of optimized tissue clearing approaches, including 3DISCO, combined with light sheet fluorescence microscopy (LSFM) and image processing permits larger volume 3 dimensional (3D) tissue analyses to reveal cytoarchitecture in greater detail^27,28^. In clearing methods, the tissue can be fluorescently stained for markers of interest and is made transparent by removal of lipids and refractive index matching with a solvent, which allows for deeper imaging of intact tissues without interference caused by light scattering through the tissue^27,28^. In LSFM, a series of images corresponding to optical sections of the tissue are reconstructed into a 3D rendering of the tissue and the large sampling volume that can be achieved in cleared tissues offers the possibility to characterize phenotypes that would otherwise be difficult to study using two-dimensional histological techniques.

Here we combined tissue clearing via 3DISCO and LSFM to image preneoplastic changes in the neonatal mouse cerebellum of Shh-MB mice. We validate that this approach can be used to quantify the effects of Norrin/Fzd4 disruption on PNL formation and EGL hyperplasia in *Ptch*^*+/−*^ and *Neurod2-Smo*^*A1+/−*^ mice, respectively. We also used this approach to perform large volume 3D imaging to show that disruption of Norrin/Fzd4 signalling is associated with spatially restricted effects on EGL hyperplasia, PNL formation and GNP migration.

## Results

### 3DISCO and LSFM reconstruction of cleared cerebella can be used to identify and quantify Norrin/Fzd4-associated changes in preneoplastic lesion formation in *Ptch*^*+/−*^ mice

**Fig. 1:**
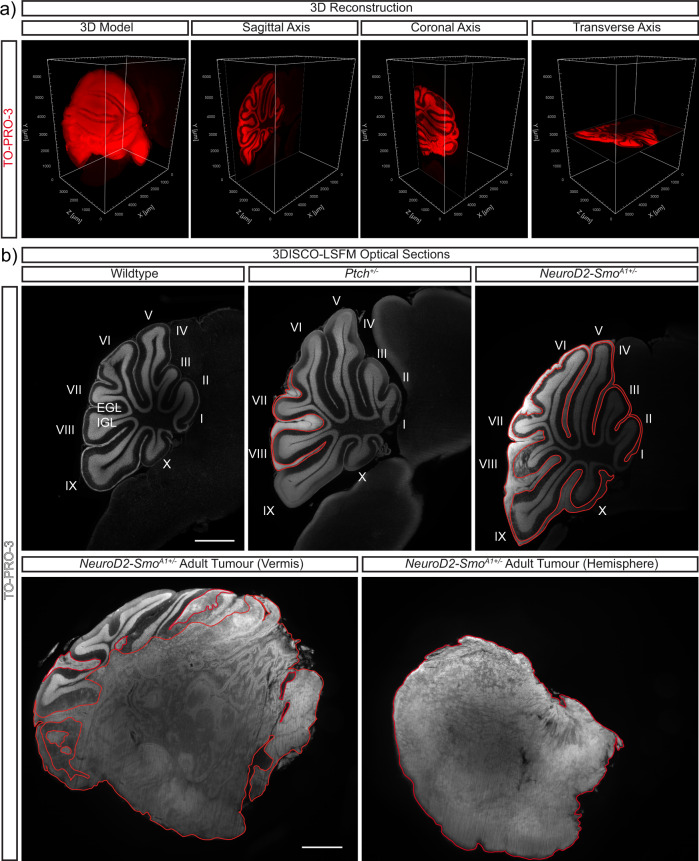
Volumetric and cross-sectional visualization of stained and cleared cerebella. **a** TO-PRO-3 (red) stained cerebellar samples viewed as 3D reconstructions of optical sections in Imaris surpass view, or as single cross-sectional images in sagittal, coronal, and transverse axes. **b** TO-PRO-3 (white) stained cerebellar samples from P14 wildtype, *Ptch*^*+/−*^, *Neurod2-Smo*^*A1+/−*^ mice and a adult *Neurod2-Smo*^*A1+/−*^ mouse with a tumour. Outlined in red: an example of a PNL, hyperplastic EGL and tumour tissue in P14 *Ptch*^*+/−*^, P14 *Neurod2-Smo*^*A1+/−*^ and the *Neurod2-Smo*^*A1+/−*^ tumour samples, respectively. Cerebellar lobes (labelled I to X) are indicated in P14 samples, scale bar: 1000 µm.

We first confirmed that we could resolve tissue architecture in 3D renderings of cleared cerebella using LSFM (Fig. 1). Macroscopic analysis of single optical sections of cleared tissue stained with TOPRO-3, a nuclear stain, in the sagittal, coronal, and transverse planes showed that the EGL and internal granule layers were well demarcated and separated by the nuclei-sparse molecular layer (Fig. 1a). Qualitative assessment of optical sections taken at the vermis revealed that when compared with wildtype mice, the cerebella of *Ptch*^*+/−*^ and *Neurod2-Smo*^*A1+/−*^ mice were larger and exhibited ectopic cell accumulations in the EGL (Fig. 1b), which is consistent with previously reported histological analyses of these mouse mutants^21,29,30^. For example, the *Ptch*^*+/−*^ cerebellum shown here contained a single PNL and the EGL in the *Neurod2-Smo*^*A1+/−*^ cerebellum is hyperplastic compared with wildtype (Fig. 1b). Additionally, it is possible to use 3DISCO-LSFM to image established MB, as shown here in adult *Neurod2-Smo*^*A1+/−*^ mouse where one can distinguish tumour and adjacent healthy tissue (Fig. 1b). Thus, LSFM imaging of TOPRO-3-stained cerebella is sensitive enough to discriminate the major layers of the cerebellum and to reveal qualitative differences in cerebellar size and EGL abnormalities in Shh-MB mutants.

**Fig. 2:**
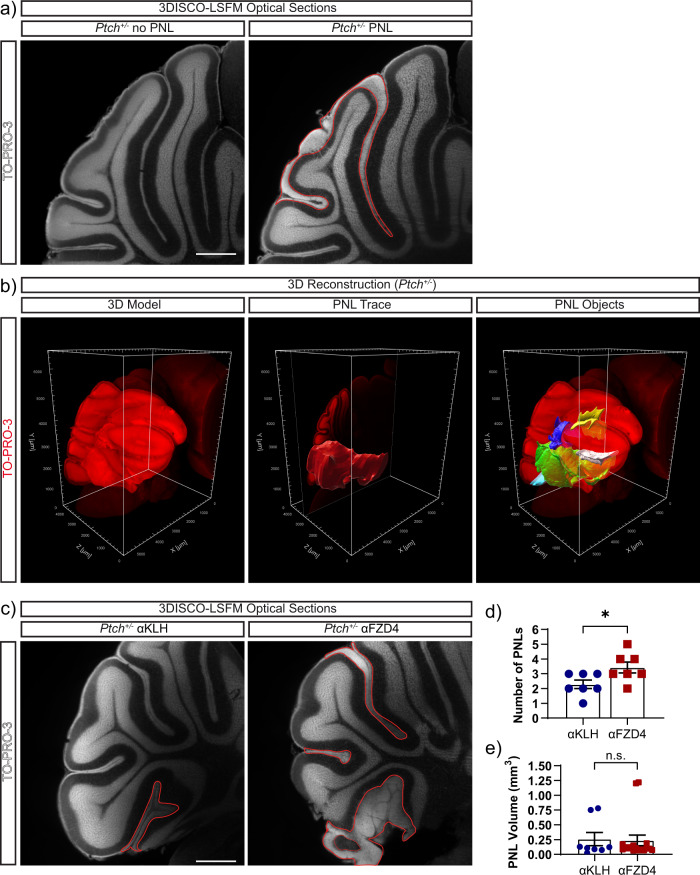
3DISCO-LSFM analysis of Norrin/Fzd4-dependent PNL formation in the *Ptch*^+/−^ cerebellum. **a** TO-PRO-3 (white) stained optical sections at the primary fissure from two different P14 *Ptch*^*+/−*^ mice. In comparison to a sample without preneoplasia, a PNL (outlined in red) displays as a distinct hyperplastic region in the EGL. **b** PNLs are identified and quantified from analysis of the 3D reconstruction of the cerebellum. TO-PRO-3 positive PNL signal is isolated from the rest of the cerebellum by manually segmenting it in 2D and combining individual traces across the sagittal axis to create a surface object representing the boundary of the PNL. A new surface object is created by masking the TO-PRO-3 channel, within the first PNL surface object, whose volume represents the true volume of the PNL. This procedure is performed for each PNL in the cerebellum (white, yellow, blue, cyan). **c** TO-PRO-3 stained (white) single optical slices showing PNLs (outlined in red) in P14 *Ptch*^*+/−*^ mouse cerebella after treatment with anti-Fzd4 (αFzd4) and anti-KLH control (αKLH) antibodies. Quantification of PNL number (**d**) and volume (**e**) in *Ptch*^*+/−*^ mice shows that the number of PNLs are increased in mice treated with αFzd4 (*n* = 7 mice) compared with control αKLH (*n* = 7 mice) antibodies. *p < 0.05, *p* = 0.0306 (unpaired two-tailed *t*-test). PNL number is reported for half of the cerebellum. **e** There was no difference in the average PNL volume in mice treated with αFzd4 (*n* = 24, PNLs) and αKLH (*n* = 11, PNLs) antibodies. n.s. *p* > 0.05, *p* = 0.4508 (Mann−Whitney test). Error bars represent s.e.m. Scale bars: 1000 µm.

Next, to determine whether 3DISCO-LSFM analysis of cleared cerebella would be as sensitive as traditional histological approaches for identifying PNLs in *Ptch*^*+/−*^ mice we developed a method to map and measure the volume of PNLs across optical sections in *Ptch*^*+/−*^ cerebella at P14. A PNL is considered a single entity if it does not overlap with another similar accumulation of GNPs in the EGL^13^. We found that PNLs were distributed on the surface of the cerebellum or deep within the folia at any point along the rostral-caudal and vermis-hemisphere axes of the tissue (Fig. 2a), which is consistent with previous studies on the histological assessments of PNL and tumour formation^16,24,31^. Next, we used this approach to detect changes in PNL formation (Fig. 2b) in *Ptch*^*+/−*^ mice treated with αFzd4 antibodies, as acute Fzd4 inhibition at P7 increases PNL formation in Ptch^+/−^ mice^24^. Cleared cerebella of αFzd4 treated *Ptch*^*+/−*^ mice (Fig. 2c) exhibited a significant increase in the number (Fig. 2d), but not the volume of the PNLs (Fig. 2e), compared to mice treated with αKLH isotype-matched control antibodies, corroborating earlier results^24^. These results demonstrate that 3DISCO-LSFM of optically cleared tissue is as sensitive as traditional histology in the quantification of PNL number and volume.

### *Ndp* deficiency induces EGL hyperplasia in *Neurod2-Smo*^*A1+/−*^ mice

**Fig. 3:**
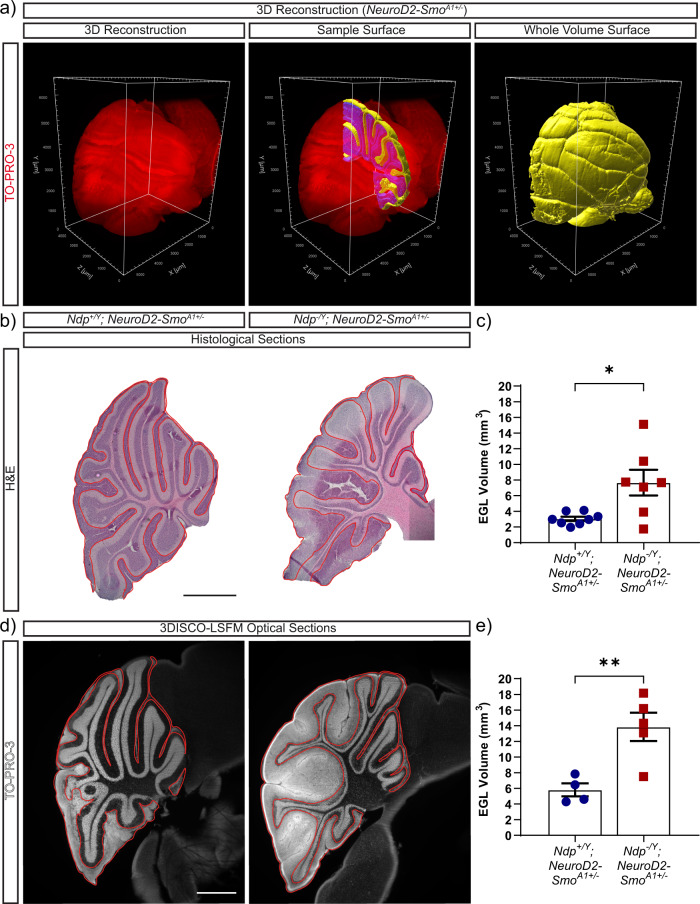
3DISCO-LSFM analysis of Norrin/Fzd4 dependent external granular layer hyperplasia in the cerebella of *Neurod2-Smo*^*A1+/−*^ mice. **a** Image processing sequence for EGL volume quantification from a TO-PRO-3 stained (red) cerebellum of a *Neurod2-Smo*^*A1+/−*^ mouse at P14. Separate EGL (yellow) and IGL (blue) surfaces from a 260 µm thick region (middle image), were generated to calculate the EGL to total (EGL + IGL) volume ratio. The EGL volume ratio was then multiplied by the volume of the entire cerebellum to calculate total EGL volume (yellow) across the sample. **b**–**e** Comparison of H&E-stained serial sections and 3DISCO-LSFM analysis of cerebella of *Ndp*^*+/Y*^*; Neurod2-Smo*^*A1+/−*^ and *Ndp*^*−/Y*^*; Neurod2-Smo*^*A1+/−*^ mice at P14. Samples are presented as H&E-stained sections (**b**), or optical sections from TO-PRO-3-stained cleared tissues (**d**). EGL hyperplasia in each section is outlined in red (**b**, **d**). **c** In H&E-stained sections, EGL volume of *Ndp*^*−/Y*^*; Neurod2-Smo*^*A1+/−*^ cerebella (*n* = 7 mice) at P14 was significantly increased compared with controls (*n* = 8 mice), *p < 0.05, *p* = 0.0106 (unpaired two-tailed *t*-test). **e** Imaris analysis of 3DISCO-LSFM samples also revealed an increase in EGL volume in *Ndp*^*−/Y*^*; Neurod2-Smo*^*A1+/−*^ mice (*n* = 5), compared to controls (*n* = 4), **p < 0.01, *p* = 0.0077 (unpaired two-tailed *t*-test). Error bars represent s.e.m. Volume measurements represent EGL volume for the entire cerebellum. Scale bars: 1000 µm.

Previously, we reported that Norrin deficiency accelerates tumourigenesis in *Neurod2-Smo*^*A1+/−*^ mice^24^, however, the impact of Norrin deficiency on preneoplasia in this mouse model was not examined. To address this question, we compared 3DISCO-LSFM with traditional histology to investigate the effect of Norrin deficiency on preneoplasia in *Neurod2-Smo*^*A1+/−*^ mice at P14. In cleared cerebella, EGL and IGL volume were quantified by segmenting regions of interest (ROIs) in the coronal plane along the rostral/caudal axis to create a single volume measurement (Fig. 3a). In manually sectioned tissue, EGL volume was estimated by combining area measurements from EGL tracings of individual sections from half a cerebellum at 120 μm intervals (Fig. 3b). Using both approaches we found that Norrin deficiency significantly increased EGL volume *Neurod2-Smo*^*A1+/−*^ mice (Fig. 3b, c). Based on these results we conclude that 3DISCO-LSFM and traditional histology are equivalent approaches to volume measurement in the EGL and that the Norrin-dependent effects on tumourigenesis in *Neurod2-Smo*^*A1+/−*^ mice are preceded by an increase in EGL hyperplasia.

### The effects of Norrin/Fzd4 signalling on preneoplasia are regionally restricted

**Fig. 4:**
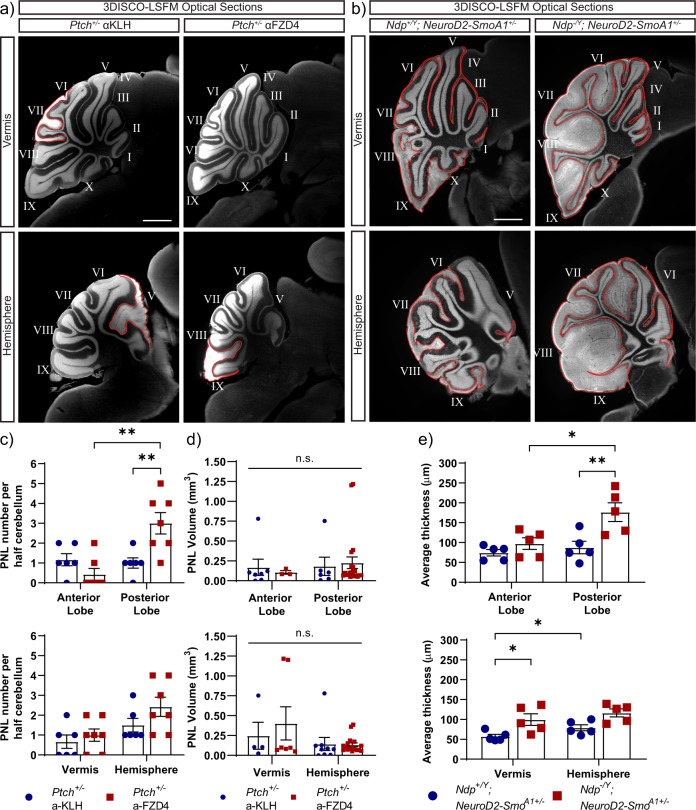
Spatially restricted effects of Norrin/Fzd4 signalling on preneoplasia in *Neurod2-SmoA1*^+/−^ mice. Representative TO-PRO-3 stained (white) optical sections of cerebella of *Ptch*^*+/−*^ mice treated with αFzd4 and αKLH antibodies (**a**) and *Ndp*^*+/Y*^*; Neurod2-Smo*^*A1+/−*^ and *Ndp*^*−/Y*^*; Neurod2-Smo*^*A1+/−*^ mice at P14 (**b**) comparing the vermis and hemisphere regions. PNLs (*Ptch*^*+/−*^) and EGL hyperplasia (*Neurod2-Smo*^*A1+/−*^) are outlined in red. Lobules of the cerebellum are labelled from I to X along the rostral-caudal axis. Number of PNLs and PNL volume in *Ptch*^*+/−*^ mice (**c**, **d**) and average EGL thickness in *Neurod2-Smo*^*A1+/−*^ mice (**e**) as a function of position. **c** PNL number is significantly increased in the posterior lobe in αFzd4-treated *Ptch*^+/−^ mice (*n* = 7 mice, αFzd4; *n* = 6 mice, αKLH) when compared to the anterior lobe of αFzd4-treated *Ptch*^+/−^ (*p* = 0.045) or the posterior lobe of αKLH-treated *Ptch*^+/−^ mice (*p* = 0.024), **p < 0.01, (Two-way ANOVA). **d** There was no spatially restricted effect on PNL volume (*n* = 4, *Ptch*^*+/−*^ αKLH, vermis; *n* = 7, *Ptch*^*+/−*^ αFzd4, vermis; *n* = 9, *Ptch*^*+/−*^ αKLH, hemisphere; *n* = 17, *Ptch*^*+/−*^ αFzd4, hemisphere), n.s. *p* > 0.05 (Two-way ANOVA); (*n* = 7, *Ptch*^*+/−*^ αKLH, anterior lobe; *n* = 3, *Ptch*^*+/−*^ αFzd4, anterior lobe; *n* = 6, *Ptch*^*+/−*^ αKLH, posterior lobe; *n* = 21, *Ptch*^*+/−*^ αFzd4, posterior lobe), n.s. *p* > 0.05 (two-way ANOVA). **e** EGL thickness was significantly increased in the posterior lobe (*p* = 0.029) and vermis (*p* = 0.0164) in *Ndp*^*−/Y*^*; Neurod2-Smo*^*A1+/−*^ mice (*n* = 4 mice) compared to *Ndp*^*+/Y*^*; Neurod2-Smo*^*A1+/−*^ mice (*n* = 4 mice). There was an increase in EGL thickness between the posterior and anterior lobes (*p* = 0.0014) in *Ndp*^*+/Y*^*; Neurod2-Smo*^*A1+/−*^ mice. In addition, there was an increase in EGL thickness between the vermis and hemisphere (*p* = 0.019) within the *Ndp*^*+/Y*^*; Neurod2-Smo*^*A1+/−*^ group. *p < 0.05; **p < 0.01 (Two-way ANOVA). Error bars represent s.e.m. Scale bars: 1000 µm.

In mouse models of sporadic Shh-MB, preneoplastic changes are more pronounced in the cerebellar hemisphere, indicating that GNPs in this region are more susceptible to the tumour promoting effects of hyperactive Hh pathway activation^31,32^. Whether other factors, including signals from the surrounding stroma play a role in the spatial regulation of tumorigenesis in the cerebellum is unknown. Thus, we compared PNL formation and EGL hyperplasia between the anterior (lobules I-V) and posterior (lobules VI-IX) regions and between the vermis and hemispheres of cleared cerebella from αFzd4-treated *Ptch*^*+/−*^ (Fig. 4a) and Norrin deficient *Neurod2-Smo*^*A1+/−*^ (Fig. 4b) mice and their respective controls at P14. Anti-Fzd4 antibody treatment significantly increased PNL number in the posterior cerebellum in *Ptch*^*+/−*^ mice (Fig. 4c) but did not affect PNL volume (Fig. 4d). To measure preneoplastic changes in *NeuroD2-Smo*^*A1+/−*^ mice, we used EGL thickness rather than volume because EGL volume can be similar between regions in the cerebellum even when there is a clear difference in thickness (Supplementary Fig. 1). Thus, EGL thickness is a better indicator of preneoplasia and can be used to compare preneoplasia between samples and between regions within the same sample. Norrin deficiency significantly increased EGL thickness and hyperplasia in the posterior lobe and vermis (Fig. 4e) of *Neurod2-Smo*^*A1+/−*^ mice. These findings indicate that the anti-preneoplastic effects of Norrin/Fzd4 signalling on preneoplasia are spatially restricted, indicating that stromal signalling contributes to regional susceptibility of GNPs to this process.

**Fig. 5:**
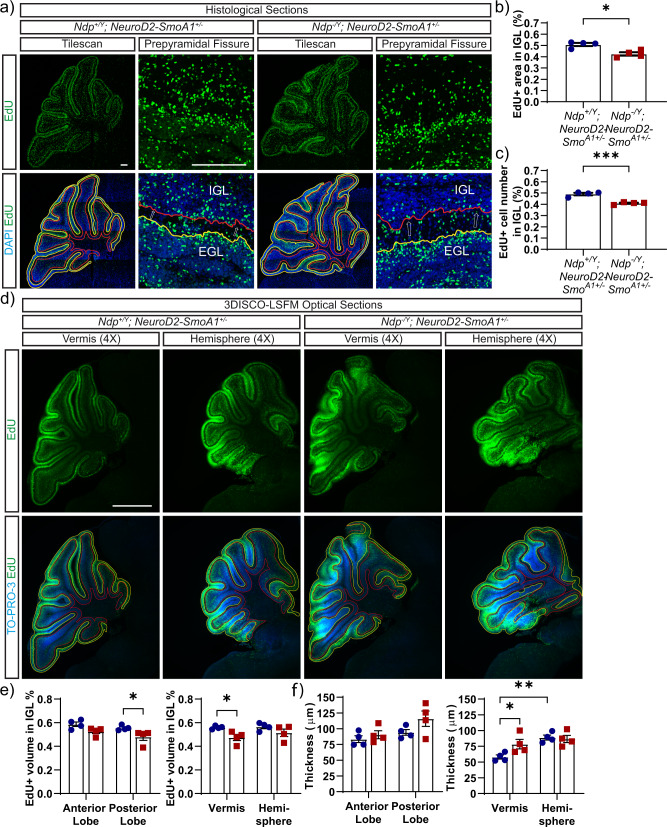
Norrin deficiency reduces granule neuron progenitor migration. **a** EdU (green) and DAPI (blue) stained histological sections of the cerebellum of *Ndp*^*−/Y*^*; Neurod2-Smo*^*A1+/−*^ and *Ndp*^*+/Y*^ *Neurod2-Smo*^*A1+/−*^ mice taken at the vermis. Shown are tilescans and an example of higher magnification of the prepyramidal fissure for each genotype. EGL (yellow outline), IGL (red outline) and direction of GNP migration (arrows). Dividing GNPs were labelled with EdU at P7 and tissues were analyzed at P9. **b** Migration index expressed as the EdU+ area in the IGL divided by the total EdU+ area in the IGL and EGL *p < 0.05, *p* = 0.0286 (Mann–Whitney test) and **c** percent of EdU+ spots in the IGL divided by the total EdU+ spots in the IGL and EGL ***p < 0.001, *p* = 0.0005 (unpaired two-tailed *t*-test) (described in “Methods”). In both cases, GNP migration to the IGL is significantly reduced in *Ndp*^*−/Y*^*; Neurod2-Smo*^*A1+/−*^ (*n* = 4 mice) compared to wildtype controls (*n* = 4 mice). **d** EdU (green) and TO-PRO-3-stained nuclei (red) in optical sections of cleared cerebellum at the vermis and hemispheres from *Ndp*^*−/Y*^*; Neurod2-Smo*^*A1+/−*^ mice and *Ndp*^*+/Y*^*; Neurod2-Smo*^*A1+/−*^ mice. **e** GNP migration was defined as the proportion of EdU+ volume in the IGL relative to the total EdU+ volume in each region. GNP migration was significantly reduced in the vermis (*p* = 0.0473) and posterior lobes (*p* = 0.0389) of *Ndp*^*−/Y*^*; Neurod2-Smo*^*A1+/−*^ (*n* = 4 mice) compared with *Ndp*^*+/Y*^*; Neurod2-Smo*^*A1+/−*^ littermates (*n* = 4 mice), *p < 0.05 (Two-way ANOVA). **f** The EGL thickness was significantly increased in the vermis of *Ndp*^*−/Y*^*; Neurod2-Smo*^*A1+/−*^ compared to *Ndp*^*+/Y*^*; Neurod2-Smo*^*A1+/−*^ controls (*p* = 0.0416). There was also an increase in EGL thickness between the vermis and hemisphere within the *Ndp*^*+/Y*^*; Neurod2-Smo*^*A1+/−*^ group (*p* = 0.0024), *p < 0.05, **p < 0.01 (Two-way ANOVA). Error bars represent s.e.m. Scale bars: 200 µm in (**a**); 1000 µm in (**c**).

Retention of GNPs in the EGL, through failure to exit the cell cycle or to migrate to the IGL, increases the likelihood of tumourigenesis, possibly through sustained exposure to pro-proliferative signals in the EGL niche^21^. Therefore, we investigated whether GNP migration is disrupted in Norrin deficient *Neurod2-Smo*^*A1+/−*^ mice. To follow GNP migration, we used a pulse-chase approach where we labelled dividing GNPs in the EGL with EdU at P7 and analyzed samples 48 h later. Because EdU incorporation is restricted to dividing GNPs on the surface of the EGL at the time of injection, the presence of EdU^+^ cells in the IGL 48 h later is due to cell migration from the EGL^21^. First, we used traditional histology to quantify the proportion of EdU^+^ cells in the IGL in the vermis of the cerebellum. In addition, to rule out effects that were based solely on differences in cell survival, the data were normalized to the total number of EdU+ cells in the EGL and IGL (Fig. 5a). We found that Norrin deficiency significantly reduced the fraction of EdU^+^ cells that migrate to IGL in *Neurod2-Smo*^*A1+/−*^ mice (Fig. 5b), suggesting that EGL hyperplasia is associated with defects in cell migration.

To examine GNP migration across a larger volume of the cerebellum, we used 3DISCO-LSFM (Fig. 5c). Consistent with our assessment of histological sections, we confirmed that GNP migration from the EGL was significantly reduced in the vermis and was also affected in posterior regions of the cerebellum in Norrin deficient *Neurod2-Smo*^*A1+/−*^ mice relative to controls (Fig. 5d). Notably, the reduction in GNP migration coincided with an increase in the thickness of the EGL in the vermis and to some extent, in the posterior cerebellum (Fig. 5e). Taken together, these results suggest that defects in GNP migration from P7 to P9 contribute to the Norrin-associated EGL hyperplasia in the vermis and posterior regions of the *Neurod2-Smo*^*A1+/−*^ cerebellum that we observe at P14. Moreover, we also validate the 3DISCO-LSFM approach to identify migration defects in the EGL.

## Discussion

Here we describe the utility of 3DISCO-LSFM for the analysis of postnatal cerebellar cytoarchitecture and preneoplasia in mouse models of Shh-MB. We demonstrate that this 3D imaging approach is as accurate as traditional histology for the identification and quantification of Norrin/Fzd4-dependent changes in PNL formation in *Ptch*^*+/−*^ mice. In addition, we show that Norrin/Fzd4 signalling in *Neurod2-Smo*^*A1+/−*^ mice regulates EGL hyperplasia and promotes GNP migration. Finally, the ability to image intact tissue shows that the anti-neoplastic effects of stromal Norrin/Fzd4 signalling are spatially restricted and localized primarily to the posterior and vermal regions of the developing cerebellum.

PNL characterization in *Ptch*^*+/−*^ mouse cerebella using traditional step sectioning^24^ involved staining and analysis of approximately 40 sections across 5 slides for each half cerebellum sample, representing approximately one tenth of the sectioned material. We estimate that this approach required 2–3 h per sample and a minimum of 2 weeks for processing and analysis of each experimental group. In contrast, 3DISCO-LSFM reduces the time required for direct sample processing, as the protocol involves a series of passive incubation steps that can be performed simultaneously on multiple samples. Thus, while the amount of time needed to clear and stain cerebella is comparable to the tissue processing for cryosections, the entire process is remarkably less laborious and can involve larger sample numbers in the same amount of time. Importantly, results from 3DISCO-LSFM analyzed cerebella were almost identical to those obtained by the traditional method^24^. An additional advantage of 3DISCO-LSFM is the higher tissue sampling volume compared with traditional histology. Thus, using the 3DISCO-LSFM approach we could more readily quantify GNP migration across the different regions of the developing cerebellum to show that Norrin deficiency was associated with reduced cell migration specifically in the posterior lobe and vermis of the cerebellum. Notably, the regions with altered GNP migration coincided with increased EGL thickness, an indicator of Shh signalling and GNP proliferation^33–35^. An important implication of these results is that direct molecular and cell biological analyses of GNPs isolated from Norrin-deficient cerebella should be conducted on cells isolated from these regions specifically.

The 3DISCO method shrinks tissue^27^; however, the shrinkage occurs evenly across the tissue and does not distort tissue architecture. However, this problem does mean that one cannot perform direct comparisons of measurements taken on cleared tissues and 2D sections. In addition, the feasibility of performing 2D histological analyses after tissue clearing has not been extensively evaluated in terms of whether de-cleared and sectioned specimens will retain normal architecture and antigen localization. Thus, a cleared cerebellum can only be used for one set of analyses, which will be limited to the number of fluorescent channels on the imaging system. Our solution to this problem is to clear half of the cerebellum, reserving the other half for 2D sections that can be analyzed for the expression of additional markers. Previous studies have demonstrated the utility of nuclear staining with Sytox Orange and RedDot2 in cleared tissue^36^ and here we extend this range of nuclear markers by showing that TO-PRO-3 Iodide and EdU are reliable markers for nuclei and cells in S-phase, respectively. It would be ideal to combine the detection of nuclei with other antigens to fully characterize lesions. In addition, the feasibility of combined staining for transcription factors and vasculature in the cerebellum has recently been demonstrated^37^. However, antigen detection in cleared specimens requires considerable optimization for each antibody. Towards that goal, we have shown that enzymatic digestion improves antibody penetration to stain ECM^36^.

In this paper, we have characterized regionally restricted effects of stromal signalling on preneoplasia in Shh-MB prone mice. Norrin deficiency increased EGL hyperplasia in the vermis and posterior cerebellum in *Neurod2-Smo*^*A1+/−*^ mice and increased PNL formation in the posterior cerebellum in *Ptch*^*+/−*^ mice. Interestingly, GNPs located in the cerebellar hemispheres, and particularly in the posterior lobes, are reported to be more sensitive to cell autonomous Hh pathway hyperactivation and exhibit an increased frequency of tumourigenesis^31,32^. Our observations corroborate these findings and suggest that regional differences in stromal signalling can contribute to the location of fully developed MB at later stages. We speculate that GNPs located in the posterior cerebellum have an increased requirement for anti-tumourigenic effects of Norrin/Fzd4 signalling in the stroma. Norrin signalling could, for example, integrate with regionally restricted genetic programmes in GNPs, such as those mediated by OTX2, which is enriched in a population of GNPs with a high proliferative capacity in the posterior lobes of the cerebellum and is required for MB progression^38^. Alternatively, the anti-neoplastic effects of Norrin signalling in the posterior stroma could be compensated by the activity of other canonical Wnt signalling components in more anterior regions of the cerebellum. Consistent with this possibility, Wnt7a-driven canonical Wnt signalling can compensate for the loss of Norrin function to establish the BBB in the cerebellum^39,40^, although whether this compensation is regionally restricted is unknown.

Disruption of Norrin/Fzd4 signalling increases neoplasia in *Ptch*^*+/−*^ and *Neurod2-Smo*^*A1+/−*^ mice^24^; however, the underlying cell biological events that drive PNL formation are unknown. Our findings suggest that defective GNP migration associated with Norrin deficiency contributes to EGL hyperplasia in *Neurod2-Smo*^*A1+/−*^ mice, which is consistent with previous studies linking defective GNP migration defects and enhanced tumourigenesis in Shh-MB in mice^21^. The EGL hyperplasia and disrupted GNP migration that we observe in the vermis of Norrin-deficient *Neurod2-SmoA1*^*+/−*^ mice are particularly noteworthy, as this region is reported to be protected from malignant progression^31,32^, and raises the possibility that the anti-tumour progression effects of Norrin/Fzd4 signalling in the stroma contribute to this regional barrier to tumourigenesis. How Norrin/Fzd4 signalling regulates GNP migration is an exciting avenue for future investigation.

In summary, our findings show that 3DISCO-LSFM is as effective and in some instances more sensitive than analysis of 2D tissue sections for the detection and quantification of pre-neoplastic phenotypes, including altered cell migration, in mouse models of Shh-MB. The methods described herein for the quantification of EGL hyperplasia and PNL development can be adapted to address related questions in CNS development and tumourigenesis.

## Methods

### Animals

All experiments were approved by the University Health Network Research Ethics Board and adhered to the guidelines of the Canadian Council of Animal Care. Animals were maintained under standard laboratory conditions and all procedures were performed in conformity with the 863 University Health Network Animal Care Committee (protocol 3499.16.2). *Ndp* knockout mice (RRID:MGI:4414648) were obtained from Lexicon pharmaceuticals and generated by disruption of the *Ndp* locus by a lacZ-containing cassette. *Ptch*^*+/−*^ mice^41^ (RRID:MGI:2177702) and *Neurod2-SmoA1*^*+/−*^ mice^12^ (RRID:MGI:3831004) were obtained from Jackson laboratories. All mice were maintained from a C57BL/6 background. Breeding pairs of *Neurod2-Smo*^*A1+/+*^ homozygous males and *Ndp*^*+/−*^ females were used to generate *Ndp*^*−/Y*^*; Neurod2-Smo*^*A1+/−*^ mice. Breeding pairs of *Ptch*^*+/−*^ males and wildtype C57BL/6 females were used to generate *Ptch*^*+/−*^ mice. In every experiment, all experimental mice (*Ndp*^*−/Y*^*; NeuroD2-Smo*^*A1+/−*^ and *Ptch*^*+/−*^ αFzd4) were compared to control mice (*Ndp*^*+/Y*^*; NeuroD2-Smo*^*A1+/−*^*, Ptch*^*+/−*^ αKLH) from the same breeding cohort to ensure matched backgrounds. Every experiment was conducted on at least 2 litters of mice on separate days, with all experimental groups present in each litter. All mice in each experiment were male. Mouse genotyping was performed by extracting genomic DNA from ear clip samples through incubation in 200 μl alkaline lysis buffer (25 mM NaOH, 0.2 mM EDTA pH 8.0) for 1 h at 95 °C. Samples were neutralized with 200 μl neutralization buffer (40 mM Tris‐HCl) and genotyped by PCR. To label S-phase cells i*n vivo*, P7 mice were injected with EdU (10 mg/mL in PBS) at a dose of 10 mg/kg intraperitoneally using a 0.3 mL insulin syringe (Cat#:8881600145, Covidien). Brains were harvested 48 h later for cell migration analysis.

### Intra cisterna magna injections

Anti-Fzd4 neutralizing antibodies and anti-KLH control antibodies were generated as described^42^ and diluted in PBS to 3 mg/mL. P7 mice were anaesthetized using cryo-anaesthesia for 15 min, immobilized and given a single injection of antibody solution at a volume of 10 µL and a dosage of 15 mg/kg directly into the cisterna magna using a 0.3 mL insulin syringe (REF:8881600145, Covidien).

### Tissue staining and clearing

Mice at P14 were euthanized by CO_2_ asphyxiation, and cardiac perfusion was performed using 5 mL of PBS, followed by dissection of the brain. The PBS perfusion step was omitted for P9 mice. Brains were cut in half at the sagittal midline and the left half cerebellum was embedded and frozen in 50:50 OCT (Sakura Finetek, #4583):30% sucrose immediately following harvesting for sectioning and histology. The right half cerebellum was fixed in 4% PFA/PBS for 2 h at 4 °C. After fixation, the samples were washed and stained in 5 mL Eppendorf tubes (Cat# 0030119401, Eppendorf). For nuclear staining, PFA fixed tissue was washed 3 times in 1× PBS at room temperature under constant agitation, permeabilized in 1× PBS with 2% Triton-X100 at 37 °C overnight with shaking, followed by incubation in 1× PBS with 2% Triton-X100 containing TO-PRO-3 Iodide (cat#:T3605, Thermofisher) (1:500 for P14, 1:1000 for P9 tissue) at 37 °C for 3 days under constant agitation. Samples were then washed in 1× PBS with 2% Triton-X100 three times for 1 h each. EdU detection in P9 tissue was performed prior to nuclear staining using the Click-IT^TM^ EdU cell proliferation kit (Cat#C10338, ThermoFisher). Briefly, P9 cerebellum samples were submerged in labelling solution overnight at room temperature with agitation. Optical clearing of TO-PRO-3 Iodide stained half cerebella was performed using the 3DISCO technique^27^. For tumour tissue, the permeabilization step was lengthened to 3 days, and the TO-PRO-3 Iodide staining step was lengthened to 7 days but otherwise the same. Samples were dehydrated using a series of methanol solutions (20, 40, 60, 80, and 100%) (Cat#6701-7-40, Caledon) for 1 h each at room temperature under constant agitation, with a final additional incubation with 100% methanol overnight. Following dehydration, samples were delipidated by incubation in a solution of 66% dichloromethane (Cat#3600-1-40, Caledon) and 33% methanol for 1 h, followed by a 15 min incubation in 100% dichloromethane. Finally, samples were immersed in 100% dibenzyl ether (Cat#33630-1L, Sigma-Aldrich), which made the sample optically clear after approximately 2 h. All incubation steps were performed in 20 mL borosilicate sample vials (Cat#GLC-04881, Qorpak). In some experiments, fixed cerebella were sectioned and processed for Haemotoxylin and Eosin staining using standard protocols, as previously described.

### Serial Sectioning and Haemotoxylin and Eosin staining

Half cerebellar samples were collected as described above from P14 mice. Samples were fixed in 4% PFA/PBS for 2 h at 4 °C. Afterwards, they were washed in PBS and incubated in 30% sucrose/PBS overnight at 4 °C. Samples were then flash frozen in 50:50 OCT:30% sucrose. Frozen samples underwent serial sectioning. Frozen samples were cryosectioned sagitally at 12 µm thickness onto Superfrost Plus positively charged slides (Fisher Scientific), dried for one hour and then stored at −20 °C. Samples were stained by Haemotoxylin and Eosin. Images for EGL volume quantification were obtained using a Canon PowerShot SD1400 IS digital camera. Representative brightfield images were taken at 5× using an Axioplan microscope and captured with an Axiocam HRc camera. Using ImageJ, the 2D area of the EGL in each section was measured from serial sections that were 144 µm apart along the mediolateral axis. The EGL area was multiplied by the difference between each serial section quantified (144 µm) to obtain an extrapolated volume for a 144 µm thick slice of tissue based on each section. The volume extrapolated from each section was added together to obtain the final EGL volume per cerebellum^24^.

### Light sheet imaging

Cleared samples were immersed in a 120 mL crystal imaging cuvette containing DBE and imaged with an Olympus MVX10 zoom microscope (LaVision BioTec, Bielefeld, Germany) using an Olympus MVPLAP 2× dry lens equipped with a LaVision BioTec solvent dripping cap. For higher magnification images, a Leica HCX APO L4X/0.95 IMM4X solvent compatible objective lens connected to an infinity-corrected zoom body (LaVision Biotec) was used. The lens was partially submerged into the DBE for focusing on the sample. The microscope was equipped with bidirectional light sheet illumination using a NKT EXW-12, Supercontinuum 1.2-watt laser and images were captured using an equipped Andor Neo sCMOS camera. The numerical aperture was 0.48 and the sheet thickness was 5 µm. Brain samples were sliced in half on the sagittal plane and oriented with the cut side face down. Only one light sheet laser was used, and the sample was oriented with the caudal side facing the laser. Each sample constituted approximately 1000–1500 optical sections. 3D reconstruction and quantification of images was done using Imaris 9.5 (Bitplane, Zurich, Switzerland).

### PNL quantification

#### Neurod2-Smo^A1+/−^ mice

EGL volume was measured in Imaris surpass view on the 3D reconstruction of the sample. The Surfaces function was used to quantify the volume of the cerebellum and its layers by thresholding for the TO-PRO-3-positive signal in the image. The volume of the EGL and IGL was quantified in three separate 250 μm thick regions of interest (ROI) spanning the coronal plane of the cerebellum. By using a surface detail of 4 µm and a background subtraction of 20 µm, we thresholded the TO-PRO-3 positive signal in the ROI and generated a volumetric object that represented the EGL and the IGL within the ROI. Once the surface object was created, any overlap between layers due to unresolvable overlap was separated manually using the Cut function. The EGL and IGL volumes within each ROI were used to determine the total volume of the entire EGL across the sample. Total EGL volume was calculated as the proportion of EGL:(EGL + IGL) from the three ROIs multiplied by the total cerebellar volume. To determine half cerebellar volume, a surface object was created encompassing the entire half cerebellum using the same parameters.

#### Ptch^+/−^ mice

PNL number was counted by scanning through the image stack in slice view. A single PNL in a P14 cerebellum was defined as a single continuous ectopic accumulation of cells in the EGL that did not overlap with any other PNLs, as previously described^13^. PNL volume was quantified in surpass view. To separate the PNL from the remainder of the cerebellum, a custom surface object was created using “Board mode”. PNL edges were traced out in two dimensions and the traces were merged into a volumetric object. This object was used to mask the TO-PRO-3 channel, which created a new channel containing only the TO-PRO-3 positive signal of the PNL. The surfaces function was then applied to the separated PNL in masked channel, using the same parameters as the volume quantifications in the *Neurod2-Smo*^*A1+/−*^ samples.

### Regional analysis of PNL location

The vermis was defined as the middle region containing all 10 cerebellar lobules and fissures, while the hemisphere was defined as any region lateral to the midline that did not contain lobules I, II, III, IX, and X. The posterior lobe and anterior lobe were separated by the primary fissure. The location of each PNL was classified as being within the anterior lobe or the posterior lobe and either the vermis or the hemisphere. Regional preferences for PNL formation were defined as the differences in the number of PNLs between each paired region per mouse.

### EGL thickness

The EGL thickness was defined as the EGL volume divided by the EGL surface area within a given ROI. For sagittal (vermis versus hemisphere) measurements, volume was measured by selecting 250 μm thick rectangular ROIs spanning the sagittal plane with one measurement at the vermis and an average taken from two measurements from the hemisphere. For coronal (posterior versus anterior) measurements volume was measured by selecting 260 µm thick rectangular ROIs spanning the coronal plane with no overlap between the posterior (lobules VI–IX) and anterior (lobules I–V) lobes. The EGL surface area was measured by tracing out the length of the EGL overlaying the cerebellum in each ROI using the measurement points function and only measuring the length from the inner side of the EGL in the ROI and measured at the 0, 125, and 250 µm mark for sagittal measurements, and at 0, 130, and 260 µm for coronal measurements.

### GNP migration

GNP migration analysis was performed on cerebella of P9 mice that were injected with EdU at P7. EdU and TO-PRO-3 volume were quantified in four regions per mouse, one each for the vermis and hemisphere (on the sagittal plane), and one each for the posterior and anterior lobes (on the coronal plane). The volume of EdU+ cells was quantified in the EGL and IGL. The ML was not quantified and was used to delimit the two granular layers. Each layer in the ROI was traced using the surfaces “Board” function in Imaris and two surface objects were created that encompassed the EGL and the IGL. The TO-PRO-3 and EdU channels were masked using the EGL and IGL surface objects, to separate each region from the whole cerebellum. The EdU positive and TO-PRO-3 positive volumes were thresholded and quantified for the EGL and IGL in each ROI. The proportion of cell migration was calculated by dividing the volume of EdU+ cells in the IGL by the total volume of EdU+ cells in the EGL and IGL. EGL thickness was measured as described for P14 *Neurod2-Smo*^*A1+/−*^ mice.

GNP migration analysis was performed on fresh frozen histological sections of EdU-treated cerebella, 12 µm step sections from the left half cerebella of mice whose right half cerebella was used in 3DISCO-LSFM GNP migration analysis. Samples were cryosectioned onto Superfrost Plus positively charged slides, dried for 1 h, and then dipped in acetone for 20 s prior to storage at −80 °C until use. Prior to staining, samples were removed from the freezer and allowed to dry at ambient temperature for 15 min. Sections were then fixed in ice cold acetone for 10 min, washed in ice cold 70% ethanol for 5 min, and then washed several times in room temperature PBS. Slides were permeabilized with 0.05% Tween-20 PBS. EdU detection was performed using the Click-IT^TM^ EdU cell proliferation kit (Cat#C10338, ThermoFisher). 10X tilescan and 20X representative images were taken using at LSM 780 confocal.

### Statistics and reproducibility

All statistical analyses were conducted using Graphpad Prism 7.0 (San Diego, CA, USA) and a *p*-value < 0.05 (denoted *) was considered significant. All means are reported with standard error of the mean. Normality testing was performed using the Shapiro-Wilk test for all comparisons between two groups. The Mann–Whitney test was performed for non-normal sample groups. For PNL counting in *Ptch*^*+/−*^ mice, Student’s t-test was used to compare the number of PNLs per individual half cerebellum between *Ptch*^*+/−*^ mice treated with αFZD4 or αKLH control antibodies. The same method was used to compare EGL volume measurements in *Neurod2-Smo*^*A1+/−*^ mice in H&E and LSFM samples. *NeuroD2-Smo*^*A1+/−*^ mice with macroscopically obvious tumours were excluded from analysis. For spatial analysis of PNL location and EGL thickness at P14, a two-way ANOVA was used with cerebellar region (vermis, hemisphere, posterior or anterior lobe) and treatment (*Ptch*^*+/−*^ α-KLH vs *Ptch*^*+/−*^ α-Fzd4) or cerebellar region and genotype (*Ndp*^*+/Y*^*; Neurod2-Smo*^*A1+/−*^ vs *Ndp*^*−/Y*^*; Neurod2-Smo*^*A1+/−*^). Statistical significance was determined using Sidak’s multiple comparisons test with repeated measures. A Mann–Whitney test was performed for changes in GNP migration measured by area, and t-test for changes in GNP migration measured by spots. For EGL thickness and GNP migration analysis in 3DISCO-LSFM samples at P9 in *Neurod2-Smo*^*A1+/−*^ mice, a two-way ANOVA was conducted using the same factors as the spatial analysis at P14. Sample size was calculated with the power analysis equation using the Z-statistic, given a two-tailed α of 0.05 and a β (type 2 error rate) of 0.2. All experiments were of sufficient power based on effect size.

### Reporting summary

Further information on research design is available in the Nature Research Reporting Summary linked to this article.

## Supplementary information

Supplementary Information

Description of Additional Supplementary Files

Supplementary Data 1

Reporting Summary

## Data Availability

The data derived from calculations used for all graphs and quantifications is compiled in the source data file Supplementary Data 1 provided with this paper. Any remaining raw data used for calculations are available from the corresponding author upon reasonable request.
