# Supplementary material for: Three dimensional reconstruction of the mouse cerebellum in Hedgehog-driven medulloblastoma models to identify Norrin-dependent effects on preneoplasia
Source: Commun Biol. 2022 Jun 9;5:569. doi: 10.1038/s42003-022-03507-5 (PMC9184598; doi:10.1038/s42003-022-03507-5)
Supplement: Supplementary file 1 — Supplementary Information [file 42003_2022_3507_MOESM1_ESM.pdf]

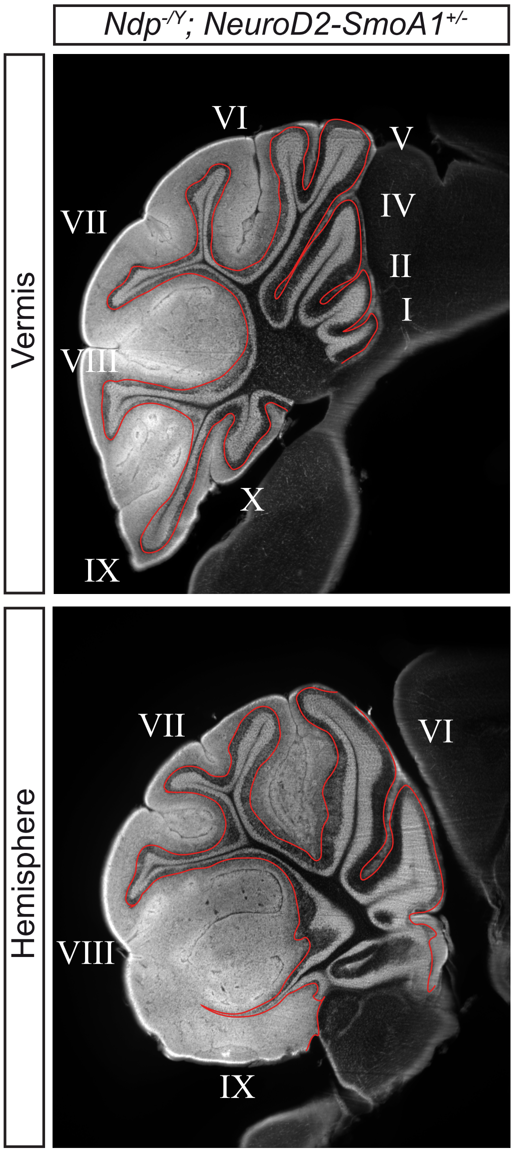

Volume = 7.8 x 10^8 μm^3
Surface Area = 8.6 x 10^6 μm^2
Average Thickness = 90.4 µm

Volume = 8.1 x 10^8 μm^3
Surface Area = 5.7 x 10^6 μm^2
Average Thickness = 143.2 µm

Supplementary Figure 1: Comparison of EGL volume, surface area and thickness between the vermis and the hemisphere of a *$Ndp^{-/Y}$; NeuroD2-$Smo^{A1+/-}$* mouse at P14. Changes in EGL volume between regions of the cerebellum do not accurately describe preneoplastic changes because of the regional differences in overall tissue size. Calculation of EGL thickness from EGL volume and surface area provides a more accurate value for the visible change in preneoplasia.
