# Supplementary material for: Three dimensional reconstruction of the mouse cerebellum in Hedgehog-driven medulloblastoma models to identify Norrin-dependent effects on preneoplasia
Source: Commun Biol. 2022 Jun 9;5:569. doi: 10.1038/s42003-022-03507-5 (PMC9184598; doi:10.1038/s42003-022-03507-5)
Supplement: Supplementary file 2 — Description of Additional Supplementary Files [file 42003_2022_3507_MOESM2_ESM.pdf]

Description of Additional Supplementary Files

File name: Supplementary Data 1
Description: The source data behind the graphs in the paper.
