# Supplementary material for: Three dimensional reconstruction of the mouse cerebellum in Hedgehog-driven medulloblastoma models to identify Norrin-dependent effects on preneoplasia
Source: Commun Biol. 2022 Jun 9;5:569. doi: 10.1038/s42003-022-03507-5 (PMC9184598; doi:10.1038/s42003-022-03507-5)
Supplement: Supplementary file 4 — Reporting Summary [file 42003_2022_3507_MOESM4_ESM.pdf]

nature portfolio

Corresponding author(s): Valerie Wallace

Last updated by author(s): Apr 26, 2022

Reporting Summary

Nature Portfolio wishes to improve the reproducibility of the work that we publish. This form provides structure for consistency and transparency in reporting. For further information on Nature Portfolio policies, see our Editorial Policies and the Editorial Policy Checklist.

Statistics

For all statistical analyses, confirm that the following items are present in the figure legend, table legend, main text, or Methods section.

n/a	Confirmed	
☐	☒	The exact sample size (n) for each experimental group/condition, given as a discrete number and unit of measurement
☐	☒	A statement on whether measurements were taken from distinct samples or whether the same sample was measured repeatedly
☐	☒	The statistical test(s) used AND whether they are one- or two-sided<br>*Only common tests should be described solely by name; describe more complex techniques in the Methods section.*
☐	☒	A description of all covariates tested
☐	☒	A description of any assumptions or corrections, such as tests of normality and adjustment for multiple comparisons
☐	☒	A full description of the statistical parameters including central tendency (e.g. means) or other basic estimates (e.g. regression coefficient) AND variation (e.g. standard deviation) or associated estimates of uncertainty (e.g. confidence intervals)
☐	☒	For null hypothesis testing, the test statistic (e.g. F, t, r) with confidence intervals, effect sizes, degrees of freedom and P value noted<br>*Give P values as exact values whenever suitable.*
☒	☐	For Bayesian analysis, information on the choice of priors and Markov chain Monte Carlo settings
☒	☐	For hierarchical and complex designs, identification of the appropriate level for tests and full reporting of outcomes
☒	☐	Estimates of effect sizes (e.g. Cohen's d, Pearson's r), indicating how they were calculated

Our web collection on statistics for biologists contains articles on many of the points above.

Software and code

Policy information about availability of computer code

Data collection	No code was used in this study.
Data analysis	Graphpad Prism 7.0 was used to conduct statistical analyses of data sets.

For manuscripts utilizing custom algorithms or software that are central to the research but not yet described in published literature, software must be made available to editors and reviewers. We strongly encourage code deposition in a community repository (e.g. GitHub). See the Nature Portfolio guidelines for submitting code & software for further information.

Data

Policy information about availability of data

All manuscripts must include a data availability statement. This statement should provide the following information, where applicable:

- Accession codes, unique identifiers, or web links for publicly available datasets
- A description of any restrictions on data availability
- For clinical datasets or third party data, please ensure that the statement adheres to our policy

The data derived from calculations used for all graphs and quantifications is compiled in the source data file provided with this paper. Any remaining raw data used for calculations are available from the corresponding author upon reasonable request.

Field-specific reporting

Please select the one below that is the best fit for your research. If you are not sure, read the appropriate sections before making your selection.

☒ Life sciences ☐ Behavioural & social sciences ☐ Ecological, evolutionary & environmental sciences

For a reference copy of the document with all sections, see nature.com/documents/nr-reporting-summary-flat.pdf

Life sciences study design

All studies must disclose on these points even when the disclosure is negative.

Sample size	Sample size was calculated with the power analysis equation using the Z-statistic, given a two-tailed α of 0.05 and a β (type 2 error rate) of 0.2. The equation used was N=((1/mean1 + 1/mean2)*(Zα+Zβ)^2)/(E/S)^2 . Where N= sample size, E= Effect size, and S= standard deviation . All experiments were of sufficient power based on effect size.
Data exclusions	In Figure 3b, mice with visible cerebellar tumours were removed from analysis. Consequently, one outlier was removed from the experimental group. No other data exclusions were performed.
Replication	Every experiment was conducted on at least 2 litters of mice on separate days, with all experimental groups present in each litter.
Randomization	For Figure 2/4, all Ptch+/- mice used were male, and samples were randomly treated with either FZD4 antibody or KLH control antibody. For figure 3/4/5, all NeuroD2-SmoA1+/- samples were male and allocated based on genotype from the same litters.
Blinding	Investigators were not blinded during sample collection or analysis. The process of sample collection is the same for all samples, and no differences between samples are apparent until sample imaging. Due to the automated nature of sample analysis, blinding was not required, as all samples were analyzed identically.

Reporting for specific materials, systems and methods

We require information from authors about some types of materials, experimental systems and methods used in many studies. Here, indicate whether each material, system or method listed is relevant to your study. If you are not sure if a list item applies to your research, read the appropriate section before selecting a response.

Materials & experimental systems

n/a	Involved in the study	
☒	☐	Antibodies
☒	☐	Eukaryotic cell lines
☒	☐	Palaeontology and archaeology
☐	☒	Animals and other organisms
☒	☐	Human research participants
☒	☐	Clinical data
☒	☐	Dual use research of concern

Methods

n/a	Involved in the study	
☒	☐	ChIP-seq
☒	☐	Flow cytometry
☒	☐	MRI-based neuroimaging

Animals and other organisms

Policy information about studies involving animals; ARRIVE guidelines recommended for reporting animal research

Laboratory animals	Mice used were from the C57BL/6 strain, all mice used were male, and samples were collected at post-natal day 9 or 14.
Wild animals	This study did not involve wild animals.
Field-collected samples	This study did not involve samples collected from the field.
Ethics oversight	All experiments were approved by the University Health Network Research Ethics Board and adhered to the guidelines of the Canadian Council of Animal Care. All procedures were performed in conformity with the 863 University Health Network Animal Care Committee (protocol 3499.16.2)

Note that full information on the approval of the study protocol must also be provided in the manuscript.
